# Prognostic Factors Associated With Mortality of Patients With COVID-19 Requiring Ventilator Management: A Retrospective Cohort Study

**DOI:** 10.7759/cureus.25374

**Published:** 2022-05-26

**Authors:** Masaatsu Kuwahara, Misa Kamigaito, Hiromoto Murakami, Kiyoko Sato, Naomi Mambo, Tomoyuki Kobayashi, Kunihiro Shirai, Atsushi Miyawaki, Munehiko Ohya, Jun-ichi Hirata

**Affiliations:** 1 Department of Emergency and Critical Care Medicine, Hyogo College of Medicine, Nishinomiya, JPN

**Keywords:** prognostic factor, mortality, critical care outcomes, covid-19, older-aged patients

## Abstract

Aim

There are few reports on the prognostic factors associated with mortality in coronavirus disease (COVID-19) patients with critical disease. This study assessed prognostic factors associated with mortality of patients with critical COVID-19 who required ventilator management.

Methods

This single-center, retrospective cohort study used medical record data of COVID-19 patients admitted to an emergency ICU at a hospital in Japan between March 1, 2020 and September 30, 2021, and provided with ventilator management. Multivariable logistic regression was used to identify factors associated with mortality.

Results

Seventy patients were included, of whom 29 (41.4%) died. The patients who died were significantly older (median: 69 years) (interquartile range [IQR]: 47-82 years) than the patients who survived (62 years [38-84 years], p<0.007). In addition, patients who died were significantly less likely to have received steroid therapy than patients who survived (25 [86.2%] vs. 41 [100%], p=0.026). In the multivariable analysis, age was identified as a significant prognostic factor for mortality and the risk of death increased by 6% for every one-year increase in age (OR: 1.06; 95% CI: 1.00-1.13; p=0.048). Medical history was not a risk factor for death.

Conclusion

Age was a predictor of mortality in critically ill patients with COVID-19. Therefore, the indications for critical care in older patients with COVID-19 should be carefully considered.

## Introduction

Approximately 14% of patients with coronavirus disease (COVID-19) develop severe symptoms, such as dyspnea, and 5% develop critical diseases requiring ventilator management [1]. In a study of over 2000 critically ill patients, age >80 years was associated with an 11-fold increased mortality risk [1,2]. Other risk factors associated with mortality from severe disease include ventilator use and the presence of comorbidities (including obesity, hypertension, and diabetes) [2,3]. Among patients with severe COVID-19 who require ventilator management, the risk factors for death are unknown, as few studies on risk factors for mortality in patients with severe COVID-19 have been restricted to patients who require ventilator management. In addition, although diabetes mellitus and other factors have been identified as exacerbating disease severity in all COVID-19 patients, the relevance of these factors among patients who require ventilator management has been questioned [4]. This study aimed to identify prognostic factors associated with mortality in patients with critical diseases who required ventilator management.

## Materials and methods

Design

This retrospective cohort study was conducted at Hyogo Medical University, a tertiary university hospital in Japan.

Patients

All patients with COVID-19 admitted to the emergency ICU between March 1, 2020 and September 30, 2021, were screened for inclusion in the study. Of the 168 patients admitted, 70 who received ventilator management were included. Patients included in the study were classified into survival and death groups.

Data collection

The following information was extracted from the electronic medical records: age, sex, sequential organ failure assessment (SOFA) score on admission, acute physiologic assessment and chronic health evaluation (APACHE) II score on admission, presence of bacteremia, medical history (obesity, asthma, chronic obstructive pulmonary disease [COPD], hypertension, diabetes mellitus, smoking history), BMI, hemoglobin A1c (HbA1c) level, presence of tracheostomy, presence of venovenous extracorporeal membrane oxygenation (VV-ECMO), the medication used (tocilizumab, steroids, remdesivir), and death.

Outcome

The primary endpoint was mortality. The secondary outcome is to identify prognostic factors involved in mortality.

Statistical analysis

Statistical analysis was performed using EZR (Saitama Medical Center, Jichi Medical University) [5]. Continuous variables of patients who died and survivors were compared using the Mann-Whitney U test and expressed as medians with interquartile ranges (IQRs). Categorical variables were compared using the χ2 test or Fisher’s exact test and were expressed as frequencies and percentages. Predictors of mortality were identified using univariate logistic regression analysis. Variables with p≤0.20 in the univariate logistic regression analysis were included in the multivariable logistic regression analysis. In all analyses, the significance level was defined as a p-value <0.05.

Ethical considerations

The study was approved by the Ethics Committee of Hyogo Medical College (registration number: 202112-058) and conformed to the provisions of the Declaration of Helsinki (as revised in Fortaleza, Brazil, October 2013). The requirement for informed consent was waived owing to the retrospective nature of this study. The report of this study complies with the Strengthening the Reporting of Observational Studies in Epidemiology (STROBE) statement.

## Results

Out of the 168 patients with COVID-19 who were admitted to our emergency ICU between March 1, 2020 and September 30, 2021, 70 received ventilator management.

Table 1 shows the characteristics of the patients who received ventilator management. The median age was 67 years (IQR: 38-84 years), and 50 (71.4%) patients were male. The median SOFA and APACHE II scores on admission were 4 (IQR: 2-13) and 27 (IQR: 9-41), respectively. In total, 30 (42.9%) patients were obese, and 6 (8.6%), 8 (11.4%), 29 (41.4%), 41 (59.4%), 27 (38.6%), and 28 (40%) had asthma, COPD, hypertension, diabetes mellitus, a history of smoking, and bacteremia, respectively. The median BMI and HbA1c levels were 23.98 kg/m2 (17.13-44.57) and 6.60 (5.5-12.5), respectively. Tracheostomy and VV-ECMO were performed in 25 (35.7%) and 10 (14.3%) patients, respectively. Tocilizumab, steroids, and remdesivir were administered to 43 (61.4%), 66 (94.3%), and 27 (38.6%) patients, respectively. Twenty-nine (41.4%) patients died.

**Table 1:** Characteristics of patients who needed ventilator treatment. APACHE: Acute Physiologic Assessment and Chronic Health Evaluation; HbA1c: Hemoglobin A1c; IQR: Interquartile range; SOFA: Sequential Organ Failure Assessment; VV-ECMO: Venovenous extracorporeal membrane oxygenation.

Factor	Value
Nuｍber (N)	70
Demographics	
Age (years), median (IQR)	67 (438-84)
Male, n (%)	50 (71.4)
Asian, n (%)	70 (100.0)
Clinical features	
SOFA score on admission, median (IQR)	4 (2-13)
APACHE II score on admission, median (IQR)	27 (9-41)
Comorbidities	
Bacteremia, n (%)	28 (40.0)
Obesity, n (%)	30 (42.9)
Asthma, n (%)	6 (8.6)
Chronic obstructive pulmonary disease, n (%)	8 (11.4)
Hypertension, n (%)	29 (41.4)
Diabetes, n (%)	41 (59.4)
Smoking history, n (%)	27 (38.6)
Body mass index (kg/m2), median (IQR)	23.98 (17.13-44.57)
HbA1c (mmol/mol), median [(QR)	6.6 (5.5-12.5)
Treatment	
Tracheotomy, n (%)	25 (35.7)
VV-ECMO, n (%)	10 (14.3)
Drug therapy	
Tocilizumab, n (%)	43 (61.4)
Steroid therapy, n (%)	66 (94.3)
Lemudecivir, n (%)	27 (38.6)
Outcome	
Death, n (%)	29 (41.4)

Table 2 shows a comparison between the patients who died and those who survived. The patients who died were significantly older than the survivors (69 years [IQR: 47-82 years] vs. 62 years [IQR: 38-84 years], p<0.007). There were no significant differences between the two groups in terms of sex (p=0.183), SOFA (p=0.569), and APACHE II (p=0.425) scores on admission, incidence of bacteremia (p=0.809), obesity (p=0.81), asthma (p=0.224), COPD (p=0.71), hypertension (p=0.806), BMI (p=0.501), HbA1c (p=0.798), tocilizumab administration (p=0.214), or remdesivir administration (p>0.999) (Table 2). However, steroids were administered significantly less frequently in patients who died than in the survivors (25 [86.2%] vs. 41 [100%], p=0.026).

**Table 2:** Comparison of death group and survival group. APACHE: Acute Physiologic Assessment and Chronic Health Evaluation; COPD: Chronic obstructive pulmonary disease; HbA1c: Hemoglobin A1c; IQR: Interquartile range; SOFA: Sequential Organ Failure Assessment; VV-ECMO: Venovenous extracorporeal membrane oxygenation.

Factor	Survival	Death	P-value
Total patients, N	41	29	
Age (years), median (IQR)	62 (38-84)	69 (47-82)	0.007
Male sex, n (%)	32 (78.0)	18 (62.1)	0.183
SOFA score on admission, median (IQR)	4 (2-10)	4 (2-13)	0.569
APACHE Ⅱ score on admission, median (IQR)	26.5 (9.0-41.0)	27.0 (18.0-41.0)	0.425
Bacteremia, n (%)	17 (41.5)	11 (37.9)	0.809
Obesity, n (%)	17 (41.5)	13 (44.8)	0.81
Asthma, n (%)	2 (4.9)	4 (13.8)	0.224
COPD, n (%)	4 (9.8)	4 (13.8)	0.71
Hypertension, n (%)	16 (39.0)	13 (44.8)	0.806
Diabetes, n (%)	24 (60.0)	17 (58.6)	>0.999
Smoking history, n (%)	16 (39.0)	11 (37.9)	>0.999
BMI (kg/m^2^), median (IQR)	22.87 (17.13-44.57)	24.46 (18.56-42.99)	0.501
HbA1c, median (IQR)	6.6 (5.7-12.4)	6.6 (5.5-12.5)	0.798
Tracheotomy, n (%)	18 (43.9)	7 (24.1)	0.129
VV-ECMO, n (%)	5 (12.2)	5 (17.2)	0.731
Tocilizumab, n (%)	28 (68.3)	15 (51.7)	0.214
Steroids, n (%)	41 (100.0)	25 (86.2)	0.026
Lemudecivir, n (%)	16 (39.0)	11 (37.9)	>0.999

Table 3 shows the results of the univariate logistic regression analysis. The univariate analysis showed a significant difference in age (OR: 1.07; 95% CI: 1.02-1.13; p=0.01) between groups.

**Table 3:** Univariate analysis of factors associated with death. APACHE: Acute Physiologic Assessment and Chronic Health Evaluation; COPD: Chronic obstructed pulmonary disease; HbA1c: Hemoglobin A1c; SOFA: Sequential Organ Failure Assessment; VV-ECMO: Venovenous extracorporeal membrane oxygenation.

Factor	OR	95% CI	P-value
Age	1.07	1.02-1.13	0.01
Male	0.46	0.16-1.32	0.15
SOFA score	0.98	0.82-1.18	0.86
APACHE Ⅱ score	1.04	0.96-1.12	0.35
Bacteremia	0.86	0.35-2.29	0.77
Obesity	1.15	0.44-3.00	0.78
Asthma	3.12	0.53-18.3	0.21
COPD	1.48	0.34-6.47	0.6
Hypertension	1.27	0.48-3.33	0.63
Diabetes	0.94	0.38-2.50	0.91
Smoking history	0.96	0.36-2.54	0.93
BMI	1.05	0.96-1.14	0.33
HbA1c	1.05	0.76-1.46	0.75
Tracheotomy	0.41	0.14-1.16	0.09
VV-ECMO	1.5	0.39-5.75	0.55
Tocilizumab	0.5	0.19-1.33	0.16
Steroids	<0.01	···	0.99
Lemudecivir	0.96	0.36-2.54	0.93

The multivariable logistic regression model included age, male sex, and tocilizumab administration (Table 4). In the multivariable analysis, age was identified as a significant prognostic factor for mortality (OR: 1.06; 95% CI: 1.00-1.13; p=0.048).

**Table 4:** Multivariable analysis of factors associated with death.

Factor	OR	95% CI	P-value
Age	1.06	1.00-1.13	0.05
Male	0.72	0.23-2.26	0.57
Tocilizumab	0.87	0.28-2.67	0.81

## Discussion

The mortality in this study was 41.4%, which was lower than that reported by Choron RL et al. [6]. They reported a mortality of 61.1% for 103 patients from the US. One of the reasons for this is the period of data collection. Choron RL et al. only collected data from March 14, 2020 to May 27, 2020, which was the beginning of the COVID-19 pandemic. However, we collected data from March 1, 2020 to September 30, 2021. The management of COVID-19 continues to evolve, and there are reports that the mortality rate is decreasing as the pandemic progresses [7-10]. The differences in the mortality rate may be explained by the decrease in mortality rate associated with the evolution of treatment as the pandemic has progressed.

In this study, age was identified as a significant prognostic factor for mortality in patients with critical diseases who required ventilator support. This result is consistent with previous reports by Choron RL et al. [6] and others [2,11]. The risk of death is high in older patients, regardless of the disease severity [1,6]. Decreased immunity may be a factor in higher mortality among the elderly. Some reports suggest that mortality may increase in the elderly because their thymus gland atrophies and T-cells decrease with age [12]. Another thing that lowers overall immunity is poor nutrition, which leads to chronic wasting disease and cancer, both of which increase with age [13]. We believe that further research is needed. The indications for critical care of older patients need to be carefully examined.

Notably, the presence of comorbidities was not a significant prognostic factor for mortality in this study. Diabetes and obesity are prognostic factors for mortality in patients with critical diseases. Comorbidities can be treated strictly, including strict insulin control for diabetes [14]. With stringent treatment, comorbidities may not be a prognostic factor for mortality. Therefore, stringent treatment of comorbidities is necessary. In this study, neither the SOFA score nor the APACHE II score on admission was a prognostic factor. One reason for this is that many patients were transferred to our hospital, and the values may have been modified due to the treatment provided prior to admission.

Treatment with Tocilizumab was also not a prognostic factor. Tocilizumab has been reported to reduce the mortality rate in two large trials, [15,16] one of which reported a benefit in patients with critical conditions. [16] However, some studies have found that tocilizumab does not improve the mortality rate. [17,18] In this study, the lack of an effect of tocilizumab on mortality may have been due to the small number of patients included in this study.

The patients who died were significantly less likely to have been treated with steroids, suggesting that steroid administration may have a positive impact on prognosis. Overall, data from randomized trials support the role of steroids in severe COVID-19 [19-21]. In a meta-analysis of seven trials including 1703 patients with severe COVID-19, steroids reduced 28-day mortality compared to standard care or placebo. Another systematic review and network meta-analysis of randomized trials evaluating interventions for COVID-19 evaluated and found that steroids were not associated with an increased risk of severe adverse events [19]. The only intervention that had at least moderate certainty of reduced mortality (OR: 0.87, 95% CI: 0.77-0.98) or risk of ventilation (OR: 0.74, 95% CI: 0.58-0.92) compared to standard care was steroids [20]. Most of the steroid efficacy data in these meta-analyses came from a large open-label trial conducted in the UK, in which 2104 and 4321 patients with confirmed or suspected COVID-19 were randomized to dexamethasone (6 mg orally or IV daily for up to 10 days) or usual care, respectively [22]. The 28-day mortality reduction with dexamethasone for the entire study population and prespecified subgroups was as follows: (1) Overall 17% relative reduction (22.9 vs. 25.7%, rate ratio [RR]: 0.83, 95% CI: 0.75-0.93); (2) Patients on invasive ventilation or ECMO at baseline; (3) Relative reduction 36% (29.3 vs 41.4%, RR: 0.64, 95% CI: 0.51-0.81). Age-adjusted analysis suggested a 12.3% reduction in mortality; (4) Patients receiving noninvasive oxygen therapy (including noninvasive ventilation) at baseline; (5) 18% relative reduction (23.3 vs. 26.2%, RR: 0.82; 95% CI: 0.72-0.94). Age-adjusted analysis suggested an absolute reduction in mortality of 4.1%. In contrast, no benefit was seen in patients who required neither oxygen nor ventilator support. In addition, there was a non-statistically significant trend toward increased mortality (17.8 vs. 14%, RR: 1.19; 95% CI: 0.91-1.55). The results were similar when the analysis was restricted to patients with laboratory-confirmed COVID-19 (89% of the total population). For patients who could be discharged before completing the 10-day course of dexamethasone, post-discharge medication continuation was not associated with further benefit [23]. Our study also suggested that steroids may have a positive prognostic impact, suggesting that it is better to continue administering steroids to patients with severe diseases.

The treatment of COVID-19 has gradually become more standardized, but at the beginning of the pandemic, each facility had a different treatment method. Therefore, the results of a single-center study such as this are limited. In addition, even within a single institution, there were differences in treatment strategies depending on the background of the patients. In addition, as this was a retrospective study, some data were not available. Another limitation of this study is the limited sample size. To overcome these limitations, it would be helpful to conduct a prospective study of patients treated using a standard treatment protocol in a multinational, multicenter setting.

## Conclusions

In patients with severe COVID-19 requiring ventilator support, age was found to be the most important predictor of mortality. Careful consideration should be given to the indications for critical care in older patients.

It is also important to strictly control comorbidities in COVID-19 patients. Strict management of comorbidities, such as strict glycemic control for diabetes mellitus, may improve the prognosis. In addition, steroids may improve the prognosis and should be administered aggressively. A prospective, multinational, multicenter study of patients treated with standard treatment protocols may be helpful in identifying more accurate prognostic factors.
